# A new transgenic mouse model for studying the neurotoxicity of spermine oxidase dosage in the response to excitotoxic injury

**DOI:** 10.1186/1750-1326-8-S1-P4

**Published:** 2013-09-13

**Authors:** Emanuela Angelucci, Sandra Moreno, Marcello D’Amelio, Roberta Nardacci, Francesco Cecconi, Paolo Mariottini, Manuela Cervelli

**Affiliations:** 1 University of “Roma Tre”, Rome, Italy; 2 IRCCS Fondazione Santa Lucia, Rome, Italy; 3 Istituto Nazionale per le Malattie Infettive, IRCCS “L. Spallanzani”, Rome, Italy

## Background

A recently generated mouse genetic model overexpressing SMO provides novel evidences of the complex and critical functions carried out by SMO and Spm in mammalian brain [1]. Spermine has been demonstrated to be the strongest PA modulator of some Glu receptors [2,3].

## Materials and methods

A mouse line was engineered with the aim to investigate the effects of SMO overexpression in brain, up to now unexplored [4]. A Cre/*loxP*-based genetic model was constructed to obtain SMO overexpressing transgenic animals conditionally. The mouse founder line (*JoSMO*), ubiquitously expressing GFP, was bred with a transgenic line expressing the Cre recombinase in the brain neocortex. Double transgenic mice (*JoSMOrec*) were obtained to specifically overexpress SMO in a tissue specific way.

## Results

*JoSMOrec* mice showed a significant astroglial and microglial activation in the neocortex, showing a more pronounced brain damage during ageing. In excitotoxic condition, KA injected *JoSMOrec* mice resulted more sensitive than control animals, showing more severe behavioural phenotype, astrogliosis and microgliosis in the neocortex and also a higher number of neurons with abnormal morphological features, all evident markers of brain injury. In SMO overexpressing mice it has been observed a different Spm/Spd ratio that could affect GluRs, producing changes in Ca^2+^ flux through GluRs and being responsible for the higher sensitivity to KA treatment [4].

## Conclusions

These results indicate an important role of SMO during excitotoxicity and neuronal damages, providing new perspectives on the complex and critical functions carried out by SMO and Spm in the physiology and pathology of mammalian brain. The production of H_2_O_2_ and AP, derived from Spm oxidation, together with direct effects of Spm on AMPA and KA receptors, are synergistically involved in ROS increase and ultimately to neuronal degeneration and death. SMO enzyme can be considered one of the most important H_2_O_2_ producers in the brain and the transgenic *JoSMOrec* mice represent a useful genetic model for studying brain pathologies such as epilepsy, Alzheimer’s disease and other forms of dementia [4].
